# PubNavigator Activity to Promote Science Argumentation in Introductory Biology

**DOI:** 10.1093/iob/obag065

**Published:** 2026-10-21

**Authors:** R A Marroquín-Flores, M A Campbell, S P Grimes, L B Limeri

**Affiliations:** Department of Biology, James Madison University, Harrisonburg, VA 22807, USA; Department of Biological Sciences, Texas Tech University, Lubbock, TX 79409, USA; Department of Biological Sciences, Texas Tech University, Lubbock, TX 79409, USA; Department of Biological Sciences, University of Rhode Island, Kingston, RI 02881, USA

## Abstract

The ability to use data and evidence as the basis for scientific argumentation is a critical skill that undergraduate students must learn to successfully transition into science careers. There is a need for pedagogical tools that promote the development of science process skills and reduce barriers to implementation in the classroom. PubNavigator (www.pubnavigator.com) is a science communication platform that shares the findings of primary literature using accessible language. Articles on the platform represent a novel form of Adapted Primary Literature accessible to novice learners. We developed a PubNavigator reading assignment that can be easily integrated into introductory-level curricula with minimal time investment from instructors. We implemented this lesson in an introductory biology laboratory and analyzed responses to an assignment prompting discussion of PubNavigator articles in an asynchronous online forum. We conducted content analysis guided by the Toulmin’s Argument Pattern to identify evidence of science argumentation and interactive engagement. PubNavigator assignments prompted students to use evidence as the basis for science arguments, evaluate experimental design, describe the purpose and relevance of scientific research, and prompted overall student engagement. Our assessment data support PubNavigator as an undergraduate teaching tool that can be used to promote science argumentation and engagement in introductory biology. We provide recommendations for implementation and possible adaptations for alternative settings and modalities.

## Introduction

Vision and Change (2011) calls for undergraduate biology education to move beyond content and to help students develop a set of core competencies that include engaging in and understanding the process of science and developing effective communication skills (American Association for the Advancement of Science 2011). The practice of science involves more than recalling a set of biological facts; students need to be able to synthesize information from various sources and use that information to construct viable explanations (Driver et al. 2000; Erduran et al. 2004). To develop these competencies, students should regularly engage in the classroom activities that prompt the development of science process skills (American Association for the Advancement of Science 2011). Learning to read and evaluate primary literature and effectively communicate research findings are widely recognized as necessary science process skills for undergraduates (Coil et al. 2010). However, there are several obstacles that prevent instructors from explicitly teaching these skills in the classroom, with time constraints being one of the largest barriers to implementation (Coil et al. 2010; Shadle et al. 2017; Petersen et al. 2020). There is an urgent need for pedagogical tools that promote the development of science process skills, and which can easily integrate into existing curricula.

Reading primary literature has been shown to promote the development of science process skills, but science students often struggle to interpret scientific literature, which can make it challenging to integrate into introductory-level classrooms (Janick-Buckner 1997; Kozeracki et al. 2006; Hoskins et al. 2007; Van Lacum et al. 2012; Krontiris-Litowitz 2013). Several tools have been developed to improve the accessibility of primary literature for introductory-level undergraduates (Round and Campbell 2013; Zagallo et al. 2016; McCartney et al. 2018; Kararo and McCartney 2019). For example, Adapted Primary Literature is a form of primary literature that uses modified language and formats to make research more digestible to novice learners, and which can be used to facilitate science discourse and support science reasoning (Falk et al. 2008; Department of Agriculture and Forestry Service 2013; Koomen et al. 2016; Ariely et al. 2019). While effective, these approaches often require instructors to invest time in reformatting the course to make room for new activities or in modifying course materials to make them more accessible to students (Falk et al. 2008; Department of Agriculture and Forestry Service 2013; Koomen et al. 2016; Zagallo et al. 2016; Ariely et al. 2019). There is a need for alternative approaches that reduce barriers to implementation.

Reading primary literature can be valuable in helping students to understand the process by which scientists use evidence to explain and understand biological phenomena. The process of scientific discovery is slow, and the evidence used to support scientific claims is continuously challenged and evaluated (Driver et al. 2000). A core facet of science involves learning to construct robust and enduring explanations (Driver et al. 2000). To participate in the process of knowledge construction, students must be able present and evaluate scientific arguments (Driver et al. 2000). An argument can be described as an assertion that uses a form of supporting or refuting evidence as justification for that assertion (Kuhn 1993). Students engaging in argumentation may do so with the goal of making sense of a new idea, articulating an idea, or persuading others of an idea (Berland and Reiser 2009). While scientific argumentation is a valuable skill to develop, students often struggle to justify claims with appropriate lines of evidence (Sadler 2004; Newell et al. 2011; Sampson and Walker 2012). However, activities that prompt students to use evidence as the basis for scientific arguments can lead to improved reasoning, conceptual understanding of the material, and metacognitive skills (Zohar and Nemet 2002; McNeill et al. 2006; Halmo et al. 2022; Ariely and Yarden 2026). Collaborative activities, where students are prompted to engage in verbal or written dialogue, can be particularly effective in helping students to strengthen their arguments and apply learned skills to new contexts (Reznitskaya et al. 2009; Newell et al. 2011; Chi and Wylie 2014; Zagallo et al. 2016). In this article, we present an accessible activity that encourages students to engage in science argumentation in an online collaborative environment.

PubNavigator (www.pubnavigator.com) is a science communication platform that shares the findings of primary literature using accessible language. The platform hosts articles that feature biographical profiles of the authors and summarize a peer-reviewed publication. PubNavigator articles are written at or below a 12th grade reading level and serve as a form of Adapted Primary Literature. Each article includes data figures and definitions for complex terms. We developed PubNavigator assignments that can be easily integrated into introductory-level curricula with minimal time investment from instructors. We selected articles to align with content in an introductory biology course, and students were asked to read and discuss the articles in an online asynchronous discussion forum as a laboratory assignment. We collected student responses to assess the extent to which PubNavigator reading assignments elicited science argumentation and engagement with the activity. We present PubNavigator assignments as practical and accessible activities that can engage students with primary literature and provide recommendations for implementation.

### Intended audience

The PubNavigator assignments are designed primarily for undergraduate students enrolled in introductory-level courses. The assignments are designed as online, asynchronous activities that students can complete as pre- or post-laboratory assignments.

### Prerequisite student knowledge

There are no prerequisites. The assignment is designed for introductory-level students and students do not need to have prior exposure to primary literature to complete the activities.

### Learning time

The assignments are designed to be completed outside of class time. Students are not timed and have 7 days to complete each assignment. However, students are expected to respond to the online, asynchronous discussion board at specified times throughout the 7-day window to better facilitate peer–peer interaction. We anticipate that each PubNavigator article will take 30–60 min to read and that crafting a written response to the assignment prompts and engaging with peers on the discussion board will take an additional 20–30 min.

### Learning objectives

Students who complete the PubNavigator assignments will:

1. Use evidence as the basis for scientific argumentation
2. Evaluate experimental design and research methodology
3. Describe the purpose and relevance of scientific research

## Procedure

This activity includes three PubNavigator readings and paired asynchronous discussion in an online discussion board. Each reading and paired discussion will take place over the course of 1 week.

### Materials

The only materials required for this assignment include access to a laptop or computer, internet access, and Microsoft Office or similar application to complete the assigned readings. The readings, assignment instructions, and grading rubrics can be found in the Supplemental Material.

### Student instructions

Students will read three PubNavigator articles featuring primary research published in academic journals (Carter 2018; Sakaluk et al. 2019; Tobin et al. 2019) (Supplemental Material, Sections 1–3). The students will be asked to discuss the articles in an online, asynchronous forum as part of a laboratory assignment. On the discussion board, students will be placed into groups of 5–7 and given a prompt to initiate discussion. Student instructions for the activity can be found in the Supplemental Material, Section 4.

### Faculty instructions

The PubNavigator reading and discussion can be completed as out-of-class assignments. We recommend dedicating a few minutes of class time to introduce the assignments and briefly explain the purpose and instructions of the assignment. We recommend that the instructor emphasizes the importance of the initial post, daily engagement on the discussion board, and authoring posts that prompt further discussion. The three articles can be assigned sequentially or spaced out across the semester. We recommend that student groups be shifted for each assignment so that students are discussing the reading with new students each time. The purpose of shifting groups is to reduce the negative effects of “lurkers,” students who are passive members of the group that benefit from the knowledge contributions of others, but who do not reciprocate by contributing to the group (Wasko and Faraj 2005; Lee et al. 2006). Lurkers can be particularly problematic in small groups, like those described here, where there are only a few members to participate in the discussion (Amichai-Hamburger et al. 2016). We found that rotating groups helped to maintain a constant flow of discussion contributors.

### Suggestions for determining student learning

We recommend assigning grades based on the timeliness of the initial post, engagement with other students, and contribution to the discussion. The grading rubric we used can be found in the Supplemental Material, Section 5.

### Sample data

Samples of student discussion board posts are included in the Supplemental Material, Section 6.

### Safety issues

There are no safety concerns related to this laboratory activity. All data collection procedures were approved by the Institutional Review Board (#IRB-2020-181).

## Discussion

### Field testing

We conducted a qualitative study to assess the effectiveness of integrating PubNavigator assignments into an introductory biology course at a public R2: doctoral-granting institution. The introductory biology course primarily enrolled freshman-level students and was designed for biology majors. Undergraduate students enrolled in the course were assigned three PubNavigator articles to read and were asked to discuss the articles asynchronously using an online discussion board. We qualitatively analyzed student discussion board posts to identify evidence of science argumentation and engagement with the activity. At the time of the study, all students were attending courses remotely due to COVID-related university closures. Lecture and laboratory sections met synchronously online once per week. During class, the laboratory sections included brief lectures on the laboratory material and discussion of weekly at-home experiments.

### Participants

Consent was obtained on a pre-semester survey. Two-hundred and fifty-seven students were enrolled in the course, and all students were expected to complete the assignments as a regular part of the course. While the majority of students completed the assigned activities, only a small subset of the students agreed to participate in the study (*n* = 23), likely due to research fatigue and reduced engagement documented during the COVID-19 pandemic (Patel et al. 2020; Wester et al. 2021). Only students who consented to participate and completed all three assignments were included in data analysis. No data were collected on students’ previous exposure to primary literature. However, the introductory biology course primarily enrolled freshman-level students, who often do not enter college classrooms with prior experience reading primary literature. Demographic data are not available for participants.

### Evidence of student learning

We analyzed the dataset using content analysis, the process of identifying common patterns within the data, and the frequency with which they occur (Srnka and Koeszegi 2007). We used a deductive approach to identify elements of an argument, as defined by Toulmin’s Argument Pattern, and an inductive approach to identify themes that emerged organically from discussion (Toulmin 2008). Toulmin’s Argument Pattern breaks an argument into a series of components that can include data, warrants, and backing in support of a given claim (Toulmin 2008). Data refer to the explicit facts used as the foundation for the claim (Toulmin 2008). Warrants are the implicit reasons (rules, principles, etc.) that justify the connection between the data and the claim (Toulmin 2008). Backing refers to facts or assumptions that support the warrant, but are not explicitly a part of the justification for a claim (Toulmin 2008). Based on this model, arguments generally take the following form: because (*data*) … since (*warrant*) … on account of (*backing*) … therefore (*claim*) (Driver et al. 2000). For example, in the hypothetical argument below, the data described are explicit results from the PubNavigator article. The warrant describes an implicit understanding of how climate change is likely to impact temperature and links the data to the claim. The backing represents the students’ interpretation of the article and provides further justification for the warrant. However, if the backing were to be removed from the argument, the data and warrant would still support the claim.

*Data*: 25% of turtles exposed to warm temperatures developed as females.
*Warrant*: Warmer temperatures are an expected outcome of climate change.
*Backing*: The article shows that more females were born at higher temperatures.
*Claim*: Climate change will lead to more female turtles.

In the deductive phase of analysis, two researchers first divided the discussion posts into identifiable units (Srnka and Koeszegi 2007). Each paragraph posted on the discussion board was initially analyzed into argumentation units, defined as a claim or series of claims and the supporting data, warrants, and/or backing used as justification for those claims. Some “paragraphs” consisted of a single sentence where a student used a carriage return to form a new line on the discussion board. Each researcher individually identified claims and the associated justification to establish an argumentation unit, then met to come to consensus on each unit. Established argumentation units were further coded to identify text used as claims, data, warrants, and/or backing. Argumentation units were first coded individually, then discussed to consensus. Only arguments that discussed the article and included some form of data (*n* = 250) were included in this phase of the analysis.

Sources of disagreement were resolved by first identifying and agreeing upon text used as claims, then identifying sources of data aligning to each claim. If data were used, but did not directly relate to a claim, and a warrant was not used to make the connection between the stated claim and data, the argument was considered incomplete. Complete arguments were further analyzed to establish the complexity of the argument, and the degree to which the argument was based on correct information. Simple arguments were defined as having a single source of data and an accompanying claim, while a complex argument was defined as having a warrant, backing, and/or several sources of data as justification for a given claim. Arguments that demonstrated an incomplete understanding of the biological topic were inductively analyzed to determine potential sources of the error.

The inductive phase of analysis involved an iterative process in which three researchers first individually coded each paragraph of text (i.e., read and identified themes present in the text, labeled as “codes” identifying the themes), then met to discuss the codes applied to each paragraph to consensus. Group discussions among the analysts would occasionally result in changes to the codebook or coding approach, in which case all previously coded data were revisited with the new approach/codebook. All qualitative data were analyzed using Delve Coding Software (https://delvetool.com/).

Upon completion of the qualitative analysis, the total number of argumentation units identified for each assignment was counted. The Kruskal–Wallis test was then used to determine if there was a difference in the number of arguments between assignments. The Wilcoxon rank-sum test was used for the *post hoc* test. The quantitative analysis was conducted using R Studio (Version 2026.07.1+147) (R Core Team 2026).

### Evidence of learning objective 1: use evidence as the basis for scientific argumentation

We identified a significant difference in the number of argumentation units by assignment (χ^2^ = 7.480, *df* = 2, *P* = 0.024). Students made significantly more arguments in the final assignment when compared to the first assignment (*P* = 0.017; Fig. 1). These findings suggest that three or more PubNavigator assignments may be necessary to solicit sufficient engagement and scientific argumentation.

**Fig. 1:**
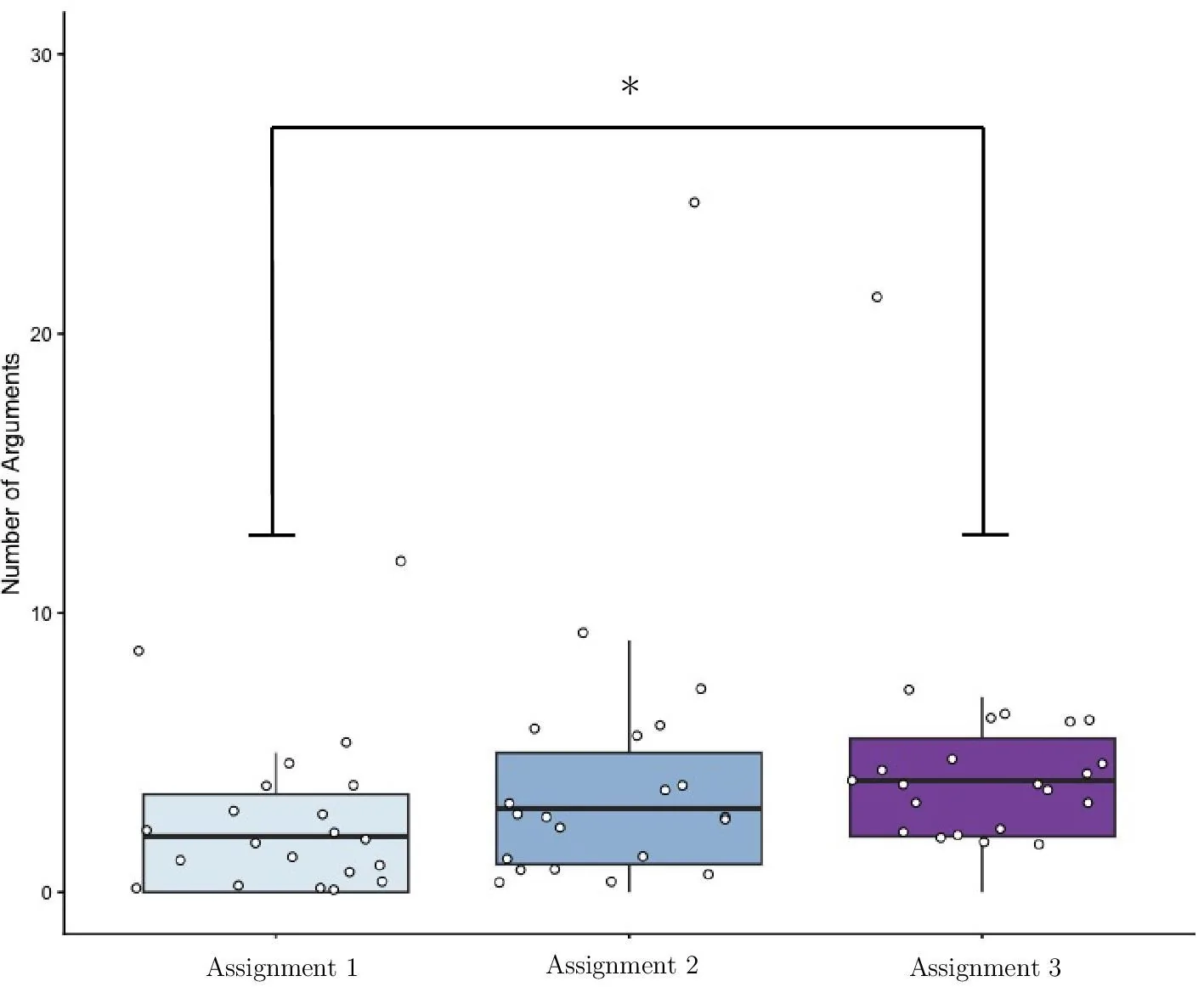
The total number of arguments that students made in each assignment: Assignment 1 = Beneficial Acclimation Hypothesis (BAH) in bumblebees (Tobin et al. 2019), Assignment 2 = inbreeding depression in crickets (Sakaluk et al. 2019), and Assignment 3 = temperature-dependent sex determination (TSD) in turtles (Carter 2018).

In the qualitative analysis, we found that nearly all students made both complete (*n* = 21) and complex (*n* = 18) arguments when discussing the PubNavigator articles. For example, in the article exploring the Beneficial Acclimation Hypothesis (BAH), the authors found that bees from different colonies had different levels of susceptibility to parasitic infection (Tobin et al. 2019). One student tried to make sense of these findings by making the following argument:

*I think that some bees were more likely to become infected than others based on what parasite was being used and their previous environments. When we think of humans, some people are more susceptible to sickness or infection based on their lifestyles at home and where they physically live, whether that be the area or their home itself. I think that bees most likely work the same way, so their ability to fight off the infection would be built up from their original habitats … Some bees may be more susceptible to one parasite than the other, and it may differ from bee colony to bee colony.*

In this example, the student claims that the bees likely experience a different environment based on the colony of origin, which can influence susceptibility to the two strains of parasites used in the study. To justify this claim, the student refers to their experiences of human illnesses (data) and suggests that bees may operate in a similar way (warrant). They further suggest that some bees may be more susceptible to the parasites used, and that this susceptibility may differ between the four colonies of bees used in the study (backing), further supporting their claim that environment and parasite strain can both influence susceptibility to infection.

While students frequently used data to justify their claims, we also found that the components of student arguments (i.e., the data, warrant, and/or backing) were sometimes incorrect, or incorrectly applied to the argument, because of an incomplete understanding of a biological concept, of the article, or of information from an external source (see full codebook in the Supplemental Material, Section 7). Across the three articles, an incomplete understanding of a biological concept was the most prominent source of error. Students often enter introductory classrooms with fundamental misconceptions about biological concepts (Coley and Tanner 2012, 2015; Stern et al. 2018). Cognitive construals refer to the informal and intuitive ways that students commonly think about the world, and which apply to a student’s understanding of biology (Coley and Tanner 2012, 2015). We found evidence of common cognitive construals, including teleological thinking and anthropocentric thinking, within student arguments.

Teleological thinking is a type of causal reasoning where students assume that there is a goal, purpose, or function to the biological concept under consideration (Coley and Tanner 2012, 2015). Students often demonstrated teleological thinking in discussions of evolution, adaptation, and mating. For example, one student wrote:

*I find your theory to be interesting and myself believe that the release of estrogen into the embryos could also be a result of evolution. I agree that the point of this evolutionary event to occur is to avoid only male turtles being born which could potentially cause extinction.*

In the article on temperature-dependent sex determination (TSD), the authors describe temporal differences in the amount of estrogen found in turtle eggs, where late-season clutches have higher concentrations of estrogen (Carter 2018). In this quote, the student demonstrates a misunderstanding of evolution. They perceive differences in yolk estrogen as a directional adaptation; the explicit purpose of having higher estrogen levels is to promote male development in late-season eggs and, therefore, to prevent the extinction of the species.

Anthropocentric thinking is a tendency to perceive humans as distinct from other animals and to make sense of unfamiliar species or biological processes by making analogies to humans (Coley and Tanner 2012, 2015). We found that students would often attribute human characteristics to the animals or use humans as a frame of reference to better understand the results of the research. For example, one student wrote:

*I think the preference [of inbred females] is due to a lot more than exposure. But I also do not discredit the role that it plays in the decision to mate with inbred or outbred crickets. I feel as though, for humans at least, we are most drawn to the people that are new and “foreign” to us over those we interact with on a daily basis. Normally humans choose to find a partner outside of their usual circle. However, it is not known if the thought process of crickets is the same or different than humans … But, perhaps the crickets know more than us and see that the inbred crickets provide them with the best chance at survival.*

In the study on inbreeding in crickets, the authors found that females from the inbred population preferred to mate with males from the same population, even though offspring from these mating events took longer to hatch and longer to develop than offspring from mating with males from the outbred population (Tobin et al. 2019). In this quote, the student is trying to make sense of these findings by making comparisons to humans. The student implies that crickets should be more attracted to mates that are dissimilar (i.e*.*, outbred) and that they intentionally select the mates that are most likely to help their offspring survive, based on the student’s perceptions of human behavior and decision-making.

The types of misconceptions identified in student discussions are often associated with types of intuitive reasoning and it is unlikely that students could resolve these misconceptions in an online discussion (Coley and Tanner 2012, 2015; Pickett et al. 2022). The PubNavigator platform contains several articles that address evolution. Given the prevalence of misconceptions regarding evolution and natural selection, it may be beneficial to pair PubNavigator assignments with activities that explicitly address common misconceptions. One option could be to integrate refutation text into lecture as a think-pair-share or into a small group discussion held during the lab. Refutation text refers to passages that include a common misconception followed by an explicit refutation of the misconception with a scientific explanation for why it is incorrect (Tippett 2010). Students who read refutation text use less language associated with cognitive construals and show improved conceptual understanding of biological evolution (Asterhan and Resnick 2020; Pickett et al. 2022).

### Evidence of learning objective 2: evaluate experimental design and research methodology

In our qualitative analysis, we identified several ways in which students were engaging with the content, including offering critiques about the experiments described in the articles (see full codebook in the Supplemental Material, Section 7). Almost all the students (*n* = 19) discussed the research methods described in the PubNavigator articles and offered a critique about how the research was conducted. For example, in the article on the BAH in bees, students often critiqued the number of variables in the study, which consisted of two strains of parasite, a range of incubation temperatures, and several colonies of bees (Chi and Wylie 2014). For example, one student said:

*I think that one of the reasons that Tobin’s data came out differently than we expected was because there were multiple strains of parasite and different colonies [of bees]. I don’t think the data would come out the same if we were to do an experiment with bees from the same colony with the same parasite. I think a change in how they respond to the parasites upon a temperature change might be different. Even if it’s the same, it will be more controlled and we can more definitively say that the results are accurate.*

The authors of the original research did not find support for the BAH, and, in this example, the student tries to make sense of the null results by identifying potential sources of error in the experimental design (Tobin et al. 2019). The student suggests reducing the number of variables (i.e., using bees from a single colony and a single strain of parasite). While this may still yield null results, the student suggests that using fewer variables would make these findings more trustworthy.

In a different discussion of the article on TSD in turtles, a student presented a possible follow-up experiment to improve the applicability of the research findings:

*One way that the experiment could be improved is to create an outside environment that can be used to test the natural temperature changes that occur in nature. Instead of recreated the temperatures in the lab. This could give more realistic results on how the eggs are affected in nature.*

In the article on TSD, the authors used a fluctuating incubation treatment to mimic the average nighttime and daytime temperatures that turtles experience (Carter 2018). However, the student suggests going one step further by incubating eggs in an outside enclosure, which would more readily capture the natural variation in temperature that real eggs experience in the wild.

It is critical for students to be able to discern the appropriateness of experimental design, to be able to interpret scientific evidence, and to question the validity of scientific claims (Erduran et al. 2004). Our findings suggest that students were regularly discussing research methodology, potentially to make sense of the research findings. Two of the articles within the assigned reading described unexpected results (Sakaluk et al. 2019; Tobin et al. 2019). When students are confronted with information that challenges their expectations or creates a sense of doubt, it can stimulate reasoning and creative problem-solving (Nussbaum and Edwards 2011; Beghetto 2017). The nature of the articles may have prompted students to engage more readily with the assignment and to identify potential sources of error in experimental design.

### Evidence of learning objective 3: describe the purpose and relevance of scientific research

Overall, we identified high levels of engagement with the PubNavigator assignments. Students regularly expressed an interest in the reading (*n* = 23), described learning new information (*n* = 21), and discussed the importance of the research (*n* = 21). Importantly, students also drew on several sources of knowledge (*n* = 22) to connect their personal experiences and their experiences in the classroom to the content presented in the articles (see full codebook in the Supplemental Material, Section 7).

Students expressing interest in the research were often surprised by the research findings, or by the ecological system being described. Students were “shocked,” “amazed,” and “blown-away” when authors could not find support for their hypotheses, or upon learning about the role that temperature can play on animal health and development. Students also clearly articulated the importance of the research being described. Students connected the research findings to their knowledge about climate change, food webs, species decline, and conservation. For example, one student said:

*I think that what was very interesting and needs to be drawn to attention more is that bees are essential pollinators in our world. They are very important to humans and many other organisms in nature. Bees are essential for the world to be the way that it is. Although the research done did not provide support for the theory being tested, it was an important experiment towards understanding the effects of humans can have on vital components of our world each and every day.*

While two of the articles included in the reading assignments described unexpected results, students were still able to understand the value of the research. Students frequently made connections between the research and their knowledge of other biological concepts learned in class, their knowledge of other species, or to their personal experiences. For example, one student used their knowledge of sex determination in mammals to try to understand the mechanism by which temperature can influence sexual differentiation in turtles:

*My understanding of genetics in turtles is very limited, but if it is anything like human genetics, another factor that could potentially be at play is the SRY gene. The SRY gene is a part of the Y chromosome that, to put it simply, makes an organism into a male. Perhaps a higher temperature could affect whether this SRY gene in males is initiated or inhibited.*

Our findings suggest that students were able to understand the relevance of teaching materials and to value the topics that were addressed within the research, both of which are related to motivation (Ryan and Deci 2000; Eccles and Wigfield 2020). Students who are able to find teaching materials relevant and meaningful are more likely to be interested in science and to perform better in their courses, and this is particularly true for students with lower expectations of success in the course (Hulleman and Harackiewicz 2009). Additionally, activities that stimulate epistemic emotions, like curiosity and astonishment, can support students’ willingness to engage in the task and motivate students to actively seek out further understanding (Vilhunen et al. 2023; Stuppan et al. 2025). The high levels of engagement suggest that these short reading and discussion assignments may be beneficial in introductory-level courses, a time during which we see a large loss of science majors (Gasiewski et al. 2012).

### PubNavigator as a form of Adapted Primary Literature

The articles included in the assignment highlighted, and were authored by, graduate researchers at the institution under study. While the topics of the articles diverged, all the articles followed a similar format and a subset of students participating in the discussion described their perceptions of the articles themselves. Students appreciated the general layout of the articles, the Decoding the Language section, which included a glossary of terms, and the introductory page containing biographical information about the author. For example, one student said:

*A couple things I liked about the article was that she added a little bit of back ground info about themselves. I also liked how it was set up overall, with each of the headings and even some figures to help understand her data even more.*

While a subset of students did discuss the biographical information included in the article, we had anticipated more comments regarding the identity of the highlighted scientist. Prior work suggests that asking students to engage with assignments that contain biographical information about a scientist can increase students’ ability to relate to the focal researcher (Schinske et al. 2016). Fourteen students made comments about the researcher highlighted in the article, but most described how passionate they thought the author was and how they imagined the author perceived their work. One student wrote:

*I think the timing of this research spotlight discussion couldn’t be more perfect as I am currently writing speeches in my communications class analyzing global warming and climate change. One thing I found interesting then and after reading this research article is that many of the effects of climate change are unknown. For that reason, I think that Dr. Amanda Wilson Carter would be excited to contribute research to such a serious topic, as would I.*

While many students often referred to the researcher’s “passion,” no students explicitly mentioned the author’s status as a student or referred to the specific hobbies or interests described in the biography. One potential explanation for these findings may be the identity of the focal researcher. Each of the articles highlighted at least one female researcher, but none of the highlighted researchers identified as a person of color, nor did they self-disclose unseen identities. It is possible that featuring counter-stereotypical examples of scientists may be more effective at improving the relatability of the highlighted researcher than featuring a near-peer (Schinske et al. 2016). However, the current layout of the articles does appear to support the accessibility of the published research. Future work can further explore the role of the biographical information on scientist relatability.

### Modifications and recommendations for implementation

Based on the assessment data, the PubNavigator assignments supported science argumentation and student engagement. Students made complete and complex arguments as they discussed the research, evaluated the experimental design presented in the articles, and described the purpose and relevance of the research. Argumentation could be further prompted by providing students with a brief description of the components of an argument and including explicit instructions to use evidence as justification for claims made on the discussion board. Instructors interested in a more in-depth approach might also consider the six-step argument-driven inquiry instructional model or a multi-stage scaffolding approach (McNeill et al. 2006; Sampson et al. 2011). Articles that address topics related to evolution or natural selection could also be paired with refutation text in lecture or laboratory as a think-pair-share, small group discussion, and/or asynchronous discussion board to avoid common misconceptions associated with these topics (Tippett 2010; Asterhan and Resnick 2020; Pickett et al. 2022). We recommend assigning three or more PubNavigator readings and paired discussions to solicit sufficient engagement and scientific argumentation.

The PubNavigator platform hosts articles from a range of science disciplines. While the assignments assessed here fit well into a life sciences curriculum, other articles could be selected using the same assignment instructions and grading rubrics. Several PubNavigator articles feature researchers traditionally underrepresented in the sciences. Instructors who are interested in helping students identify with scientists could also select articles based upon these featured researchers. Finally, while the articles described here were implemented as an asynchronous online laboratory assignment, this assignment could be easily converted into an in-person discussion.

## Conclusion

The ability to use evidence to support scientific claims sets the foundation for scientific thinking (Driver et al. 2000). We have used articles from the PubNavigator platform to develop a low-stakes assignment that can be easily integrated into introductory-level biology courses. The assignments encourage students to practice science argumentation and motivates student engagement. In our assignment, we asked students to discuss their reading in an online forum, and students were graded based on engagement and contribution to the discussion. The assignment instructions and assignment structure were designed to encourage students to interact daily. We analyzed student responses to the assignment and found evidence that the assignment successfully elicits scientific argumentation among undergraduates in an introductory biology course. PubNavigator reading assignments can be selected to fit within the curriculum and can easily be integrated into an online or in-person laboratory associated with the course.

## Supplementary Material

obag065_Supplemental_File

## Data Availability

The qualitative data underlying this article are available in the online supplementary materials. The quantitative data and program code underlying this article are available on OSF: osf.io/u7dsa.
