# Supplementary material for: PubNavigator Activity to Promote Science Argumentation in Introductory Biology
Source: Integr Org Biol. 2026 Oct 21;8(1):obag065. doi: 10.1093/iob/obag065 (PMC13736555; doi:10.1093/iob/obag065)
Supplement: Supplementary file 1 [file obag065_supplemental_file.docx]

**Supplemental Materials for:**

**PubNavigator activity to promote science argumentation in introductory biology**

**Corresponding author:** Rosario Marroquín-Flores ([marroqra@jmu.edu](mailto:marroqra@jmu.edu)), Department of Biological Sciences, James Madison University

**Table of Contents**

**Section 1:** Reading Assignment #1: “Infection outcomes not significantly	Page 2 – 8

affected by thermal variability in a bumble bee host-parasite system”

**Section 2:** Reading Assignment #2: “Effects of Inbreeding in Crickets”	Page 9 – 15

**Section 3:** Reading Assignment #3: “Turning up the heat: how turtle	Page 16 – 20

hatchlings respond to environmental temperature”

**Section 4:** Student Instructions	Page 21

**Section 5:** Grading Rubric	Page 22

**Section 6:** Sample Discussion Board	Page 23 – 24

**Section 7:** Codebook for Qualitative Assessment	Page 25 – 27

**Infection outcomes not significantly affected by thermal variability in a bumble bee host-parasite system**

**Featured Scientist:** Kerrigan B. Tobin, M.S. 2019, School of Biological Sciences, Illinois State University

**Birthplace:** Normal, IL

**My Research:** I study how **climate change** influences bumble bee immune function and survival.

**Research Goals:** I want to continue to use a combination of laboratory and field techniques to address **native pollinator** conservation. I think it’s important to understand how human impacts on Earth may have an effect on the organisms around us.

**Career Goals:** I’m currently a lab technician for the USDA, but eventually I’d like to be an Ecology professor, where I can teach and mentor students from various backgrounds about fascinating relationships in nature and how we can support biodiversity.

**Hobbies:** Crafts, especially throwing pottery and crocheting.

**Favorite Thing About Science:** It’s so satisfying to find evidence and support for a theory!

**Organism of Study: *Bombus impatiens***, the common Eastern Bumble Bee

The Eastern bumblebee, ***B. impatiens***

**Field of Study:** Ecology

**What is Ecology?** The study of how living organisms interact with each other and their surroundings. It’s an interesting field because we can study ecology at different scales. We can look at how a single organism interacts with its environment or study how complex networks of species interact in a community.

**Check Out My Original** [**Paper**](https://academic.oup.com/icb/article-abstract/59/4/1103/5486595)**:** “Infection Outcomes are Robust to Thermal Variability in a Bumble Bee Host–Parasite System”

*QR Code to original publication*

**Citation: K.B. Tobin**, A.C. Calhoun, M.F. Hallahan, A. Martinez, B.M. Sadd, Infection Outcomes are Robust to Thermal Variability in a Bumble Bee Host–Parasite System. *Integr. Comp. Biol.* 59.4: 1103-1113 (2019).

**Research at a Glance:** In this paper, we wanted to test whether changes in temperature make it more difficult for a bumble bee to fight off infections. **Climate change** is predicted to make temperatures more variable. These changes are likely to have an impact on many animals and how they interact with their environments. Living creatures have an optimum range of temperatures where their bodies perform the best. Outside of this range, their bodies cannot carry out important tasks. These ideas suggest that **climate change** might hurt animals because they will have to survive outside of the range of temperatures they are used to. Some scientists explain this using the “**beneficial acclimation hypothesis**” or **BAH**. This idea suggests that animals adjust to their environment, which gives them advantages for survival. For example, animals that live in the desert are particularly good at surviving in desert conditions. But, animals from the arctic are bad at surviving in the desert. On the same line of thought, we wanted to know if bumble bees were better at fighting off infections when they were living at the temperature that they were used to, their **acclimation temperature**. We wanted to see if bees living in a temperature outside their normal range were more likely to get parasites than bees kept at their normal temperature. To test this, we used the Common Eastern Bumble Bee as a host and a gut parasite that often infects bumble bees, ***Crithidia bombi***.

In our experiment, we used bees from four different colonies. Each bee was given one week to acclimate, or get used to, a specific temperature. They were acclimated to 21°C, to room temperature (between 25-26°C), or to 29°C. Next, we gave each bee one of two strains of the parasite, ***C. bombi***. This parasite is commonly found in the gut of the bumble bee. We gave the bees the parasite by mixing ***C. bombi*** into sugar water that was fed to them. After parasite exposure, bees were then put in a **performance temperature** of 21°C, room temperature (between 25-26°C), or 29°C. This means that some bees went back to the temperature that they were acclimated to, but others were placed into a different, **mismatched**, temperature. At four and six days after parasite exposure, we checked to see if the bees were infected by collecting their feces and looking for parasite cells inside. Eight days after parasite exposure, we froze the bees and measured how intense the infection was.

We found that the strain of ***C. bombi*** matters, because the two strains of parasites did not infect bees at the same rate. We also found that bees from different colonies had a different susceptibility to infection. This means that some bees are more likely to get an infection based on their colony of origin. Finally, we found that **constant** and **mismatched** temperatures did not impact whether the bees became infected or how intense an infection became. These results mean that we did not find support for the **BAH**. Overall, our results suggest that small changes in temperature are not changing the relationship between bumble bee hosts and their parasites. We also showed that some parasites are better at infecting hosts than others, and some colonies are more likely to get infected than others. More work must be done to really understand how **climate change** might impact bee health, but our study is a good first step.

**Highlights:** The goal of this research was to test the **BAH** in the bumble bee host-parasite system. The **BAH** says that bees used to one condition, but given an infection with a parasite in different conditions, would have worse outcomes than bees that stayed in **constant** conditions. We tested this by keeping bees in **constant** temperatures or letting them get used to one temperature and then moving them to a new temperature. Then, bees that were kept at **constant** temperature and bees that experienced **mismatched** temperature were each given a dose of parasites. We screened the feces of each bee at 4 and 6 days after parasite exposure. We measured the intensity of the infection eight days after exposure to the parasite by performing **qPCR** on the gut of each bee. **qPCR** is a laboratory method that allows us to estimate the number of parasite cells contained in each bee.

We compared temperature treatments and infection over time to analyze our results. **Figure 1** shows the number of bees that had parasite cells in their feces 4 days after they were infected. On day 4, there was no difference between bees that were kept at **constant** or **mismatched** temperatures.

**Figure 1.** Infected bees 4 days after ***C. bombi*** exposure. The x-axis shows the temperature treatment, and the y-axis shows the proportion of bees that became infected. *Adapted from Tobin et al. 2019.*

**Figure 2** shows the number of bees that had parasite cells in their feces 6 days after they were infected. On day 6, more bees were infected, but there was still no difference between bees that were kept at **constant** temperature and those that experienced a **mismatched** temperature.

**Figure 2**. Infected bees 6 days after ***C. bombi*** exposure. The x-axis shows the temperature treatment, and the y-axis shows the proportion of bees that became infected. *Adapted from Tobin et al. 2019.*

**Figure 3** shows the intensity of the infection 8 days after the bees were infected. There was no difference between bees that were kept at a **constant** temperature and those that experienced a **mismatched** temperature.

**Figure 3.** Intensity of ***C. bombi*** infection 8 days after bees were exposed to the parasite. The x-axis shows the temperature treatment, and the y-axis shows a measurement of infection intensity. The data was **log-transformed** to make it easier to interpret. *Adapted from Tobin et al. 2019.*

Taken together, **Figure 1-3** show that temperature did not change whether or not a bee was infected with the parasite or how intense the infection was. This means that we did not find support for the **BAH** in the bumble bee host-parasite system within the range of temperatures that we tested. More research needs to be done to understand the impact of **climate change** on this and other systems.

**What My Science Looks Like:** The methods that I used in my experiment are presented in **Figure 4**. The boxes on the left show the temperature that a bee experienced during the 7-day **acclimation** period. After this, the bees were exposed to one of two strains of the ***C. bombi*** parasite. After exposure, bees were either returned to their original temperature (long dashed black arrows, **constant**), or they were assigned to a different temperature (short dashed gray arrows, **mismatched**). The boxes on the right side of **Figure 3** show the **performance temperature**. We quantified **transmission** 4 and 6 days after parasite exposure and we quantified the intensity of the ***C. bombi*** infection 8 days after parasite exposure.

**Figure 4**. Breakdown of the methods used for the described experiments. Bees started in one temperature (left), were exposed to parasites (center), and were moved to a new temperature before being sampled (right). *Adapted from Tobin et al. 2019.*

**The Big Picture:** Human-driven **climate change** has many effects on earth, but its full range of impacts are not well understood. This research looked at how one part of **climate change**, thermal variability, will influence a specific host- parasite system. Although we didn’t find support for the hypothesis that we tested, other effects of **climate change**, like prolonged exposure to high temperature or heavy rainfall, may impact bumble bees and other pollinators. Humans need pollinators to help create the food that we consume, but we are not the only living things that rely on them. Many ecosystems need pollinator services to help plants thrive and produce the fruits that feed many other animals. Pollinator species are in decline and we cannot help them until we understand why it’s happening. Research like mine is important because it helps us to see why pollinators are disappearing. Our work will help to focus conservation efforts.

**Decoding the Language:**

**Acclimation temperature:** The temperature that an organism adjusts to over time. In the context of this experiment, this was the temperature that bees had 7 days to get used to before we introduced parasites and tested them.

**Beneficial acclimation hypothesis (BAH):** The BAH is a hypothesis that suggests that animals that have lived in a particular environment for a long period of time have adapted to that environment. These animals are particularly suited to this environment and will survive better than animals that are from somewhere else.

***Bombus impatiens (B. impatiens)*:** The scientific name for the common Eastern Bumble Bee.

**Climate change:** A change in global or regional climate patterns, often seen as major changes in temperature or precipitation.

***Crithidia bombi* (*C. bombi*):** The scientific name for the gut parasite that was used to infect bees in this study.

**Constant:** The bees that started in one temperature and were shifted to the same temperature later in the experiment experienced a constant temperature.

**Log-transformed**: When data are in a format that is hard to analyze or show graphically, scientists can scale their data set by performing a mathematical operation, like a logarithm, to scale the data.

**Mismatched:** The bees that started in one temperature, but were shifted to another temperature later in the experiment experienced a mismatched temperature.

**Native pollinator:** “Pollinator” refers to any animal or insect that helps to carry pollen from flower to flower. A native pollinator is an animal that provides pollination services to flowers and is local to the area. For example, bumble bees are native pollinators because they naturally occur in the United States. The honey bee is not a native pollinator because these bees were imported into the country.

**Performance temperature:** In the context of this experiment, the performance temperature was the temperature that the bee was experiencing when we tested for the effects of the parasite infection.

**Quantitative polymerase chain reaction(qPCR)**: qPCR is a lab technique where known quantities (or standards) of DNA are amplified at the same time as unknown DNA samples. It is used to estimate the DNA concentration in each unknown sample based on how much the unknown samples were amplified relative to the standards.

**Transmission:** Transmission refers to whether or not the bee became infected with the parasite.

**Learn More:**

Pollinator [Partnership](https://www.pollinator.org/)

U.S. Department of Agriculture pollinator [page](https://www.ars.usda.gov/pacific-west-area/logan-ut/pollinating-insect-biologymanagement-systematics-research/), Logan, UT (This is where I work!)

Pollinator Conservation Resource [Center](https://xerces.org/pollinator-resourcecenter)

The Intergovernmental Panel on Climate Change (I[PCC](https://www.ipcc.ch/))

*Synopsis edited by: Elyse McCormick, M.S. (Anticipated Spring 2022) and Rosario Marroquin-Flores, PhD (Anticipated Spring 2022), School of Biological Sciences*, *Illinois State University.*

**Effects of inbreeding in crickets**

*Illinois State University dual contributor*s.

**Featured Scientist:** Kylie Hampton, M.S. 2020, School of Biological Sciences, Illinois State University

**Birthplace:** Chicago, IL

**My Research:** I am interested in understanding how the female immune system is impacted by mating in decorated crickets.

**Research Goals:** I hope to continue to work in the field of Behavioral Ecology because it provides the opportunity to ask really exciting research questions. I am specifically interested in mating behavior and the study of the insect immune system. However, I have diverse research interests and would be excited to gain experience in other fields as well!

**Career Goals:** I hope to be a research assistant in a lab that has similar research interests. I really enjoy doing lab work involving microscopy and microbiology but I’d be excited to give field work a try, too!

**Hobbies:** I love listening to true crime podcasts, drinking wine, reading, and painting!

**Favorite Thing About Science:** I love science because it’s an entire field devoted to understanding the world. Every project that I have been a part of has been my personal attempt to contribute something new or to support existing knowledge, which I find to be a really rewarding experience.

**Featured Scientist:** Ian Rines, PhD (Anticipated Spring 2023), School of Biological Sciences, Illinois State University

**Birthplace:** Charleston, SC

**My Research:** Male crickets produce **compounds** that they feed to females during mating. I study how male crickets use these **compounds** to manipulate female behaviors during and after reproduction.

**Research Goals:** I’m interested in the study of animal behavior and am broadly interested in continuing to work with insects. I would like to use **molecular techniques** to change how genes are expressed in crickets. These techniques should help me to understand what causes certain behaviors.

**Career Goals:** I’m not entirely sure what I’d like to do in the future, but right now, I’m hoping to do research in an academic setting.

**Hobbies:** I enjoy reading, watching movies, and (mild) hiking.

**Favorite Thing About Science:** My favorite thing about science is actually doing the science itself. It’s fulfilling to design and carry out experiments to get at an answer to a question.

**Organism of Study:** We study the decorated cricket, ***Gryllodes sigillatus***.

***G. sigillatus****, or the decorated cricket. Pictured are a male (left) and a female (center). This pair has just finished mating, and the female is bending around to remove the gift (the clear capsule she is grasping).*

**Field of Study:** Behavioral Ecology

**What is Behavioral Ecology?** Animal behavior is shaped over time based on ancestry and environmental surroundings. Good behaviors, those that help animals to survive, become more common. Bad behaviors, those that do not help animals survive, become less common. Behavioral ecology is the study of how these behaviors are formed.

**Check Out My Original** [**Paper**](https://www.sciencedirect.com/science/article/pii/S0003347219302222?casa_token=-VFAgIyx36QAAAAA:VdxFWyfRFNce7qDmSxZaOeBXl7nJBVc5tbmCowuCxZbD6kLlbcNcqfAROvJfeGH7d22QzDq3Qr4)**:** “Effects of inbreeding on life-history traits and sexual competency in decorated crickets”

*QR code to original publication*

**Citation:** S.K. Sakaluk, J. Oldzej, C.J. Hodges, C.L. Harper, **I.G. Rines**, **K. J. Hampton**, K.R. Duffield, J. Hunt, B.M. Sadd, Effects of inbreeding on life-history traits and sexual competency in decorated crickets. *Anim. Behav.* 155: 241-248 (2019).

**Research at a Glance:** This paper presents the results of two undergraduate research projects from several years ago. We helped to write the results of their work for publication. We studied how **inbreeding** affects mating and offspring production in decorated crickets. The crickets were originally collected in New Mexico in 2001. They were mated several times to siblings to create genetically distinct lineages of crickets. To see the impact of extreme **inbreeding**, we measured the number of offspring that the female crickets produced. Not surprisingly, we found that **inbred** crickets had fewer offspring. Here, **inbred** crickets were from the lineages that experienced **inbreeding**. These offspring took longer to hatch and grow into adults. We also did another experiment to see how **inbreeding** impacts **mating success**. From that study, we found that **inbred** males are less successful at completing their steps in mating, which involve attaching a **sperm package** to the female’s genitalia. Surprisingly, we found that inbred females preferred **inbred** males from within their own line, that is, the males that were most closely related to them. This result seemed to contradict all the previous results found in the literature, which indicated that **inbreeding** had negative effects in these crickets. In general, **inbreeding** is considered a bad strategy for reproduction, as it can lead to **inbreeding** depression. **Inbreeding** depression is a reduction of **reproductive output** due to mating between relatives.

**Highlights:** Experiment 1: In the first experiment, we examined the effects of **inbreeding** on the offspring. We looked at hatching and development times, the number of offspring produced, the offspring size, and various other outcomes of mating. In **Figure 1**, we compared the number of days that it took to hatch for **inbred** and **outbred** crickets. Here, **outbred** crickets are from lineages that did not experience **inbreeding**. We found that **inbred** crickets take longer to hatch.

**Figure 1.** Hatching time of **inbred** and **outbred** offspring. The x-axis shows whether the crickets were **inbred** or **outbred**, while the y-axis shows the average number of days that crickets took to hatch. Hatching time was the time from when the egg was laid to when the cricket emerged from the egg. *Figure adapted from Sakaluk et al.2019.*

In **Figure 2,** we compared the number of offspring that were produced from **inbred** and **outbred** crickets. We found that **inbred** crickets have fewer offspring than **outbred** crickets.

**Figure 2.** Number of offspring from **inbred** and **outbred** matings. Again, the x-axis shows whether the crickets were **inbred** or **outbred**. The y-axis is the average number of offspring produced by females. *Figure adapted from Sakaluk et al. 2019.*

In **Figure 3**, we looked at the amount of time that it took male and female offspring to develop into adults because sex is known to affect development time. We then compared development time for **inbred** and **outbred** crickets. We found that, regardless of sex, **inbred** crickets took longer to develop into adults. Overall, these results show that **inbreeding** can have negative impacts on the cricket offspring.

**Figure 3.** Development time of **inbred** and **outbred** offspring by sex. The x-axis shows the sex of the crickets, while the y-axis indicates the average development time in days. Development time was characterized by how long it took crickets to reach sexual maturity. *Figure adapted from Sakaluk et al. 2019.*

Experiment 2: Our second experiment, where we studied the effects of **inbreeding** on mating success, yielded very surprising results. We observed the behaviors of several cricket mating pairs. We paired **inbred** males with **inbred** females and **outbred** males with **outbred** females, then watched them mate. Next, we paired **inbred** males with **outbred** females and **outbred** males with **inbred** females. We did this to make sure that we had all possible mating combinations (**Table 1**). To observe mating, we placed males and females into small plastic containers in a dark, warm room. We had the room illuminated by red light because crickets can’t see red light and it allowed us to observe them.

**Table 1.** Layout of all mating combinations we observed.

To successfully mate, male crickets must attach the **sperm package** to the female. We found that **inbred** males had a lower chance of successfully attaching the **sperm** **package**, regardless if they were paired with **inbred** or **outbred** females. We think that **inbred** males are just bad at mating. This is because previous research shows that males are more likely to experience the negative effects of **inbreeding** when it comes to the steps in mating.

Surprisingly, **inbred** females were more likely to mate with **inbred** males. Given these results, we cannot simply conclude that “**inbreeding** is bad”, because **inbred** females seem to prefer mating with **inbred** males. While our experiments don’t tell us why this is happening, we have several ideas. Our **inbred** lineages of crickets have been isolated within their **inbred** lines from a long time. It is possible that females don’t recognize males from outside of their lineages. They may not see these outsider males as acceptable mates because they are only ever exposed to one type of male, that is, their **inbred** counterparts.

**What My Science Looks Like:** The image the below is a mating chamber. We illuminate the room in red light, then observe the mating behavior through this observation chamber.

*Image of a mating observation chamber.*

**The Big Picture:** Studying the effects of **inbreeding** in crickets may not seem like an important thing to research. Our cricket species is not endangered or declining, but the same cannot be said for many other insect species. Substantial declines have been reported in bees, moths, butterflies, dragonflies, and other insects. There are approximately 1.5 million species on our planet, and insects account for more than half of those species. Compared to most vertebrates, it is much harder to determine whether an insect species is declining. When species go into decline, they are more likely to experience **inbreeding**, and this can negatively impact the species. **Inbreeding** can harm offspring by shortening their lifespans, damaging their health, and reducing their **reproductive output**. Given these negative effects, we would expect **inbred** individuals to avoid mating each other. However, our research shows that animals may not always avoid **inbreeding**. This is particularly important because we also find that **inbred** males are less successful at mating. Our study has led to surprising results and further unanswered questions. Future research should help to unravel these mysteries and focus on the effects of **inbreeding** on multiple traits (such as mating), not just **life history traits**.

**Decoding the Language:**

**Compound:** A chemical compound is made up of two or more elements. For example, table salt (NaCl) is a chemical compound because it is made up of both sodium (Na) and chlorine (Cl).

***Gryllodes sigillatus (G. sigillatus)*:** The scientific name for the decorated cricket.

**Inbreeding:** Reproduction between closely related animals.

**Inbreeding depression:** The reduction of reproductive output due to mating between relatives.

**Life history traits:** Traits that may influence the fitness of an organism, specifically those involved in growth, survival, and reproduction.

**Mating success:** In the context of our study, mating success refers to the successfully completing all the steps in copulation, starting with a male courting and a female terminating sperm transfer.

**Molecular techniques:** A method used to manipulate and understand DNA, RNA, or protein.

**Outbred:** In the context of this study, these are crickets that were not subjected to full-sibling matings and were allowed to mix freely.

**Reproductive output:** The number of offspring produced by a female.

**Sperm package:** To mate, male decorated crickets will attach a sperm package to the genitalia of the female. The package contains a combination of proteins that the female will feed on after it is attached to her body. As she feeds, sperm from the package will enter the female’s reproductive tract. The longer the package is attached to her body, the more likely it is for the female to be fertilized by the male’s sperm. It is in the male’s best interest to successfully attach the package and to make it as tasty as possible. This lengthens the amount of time that the female feeds and the amount of time that the sperm has access to her reproductive tract.

**Learn More:**

Insect [decline](https://www.nationalgeographic.com/animals/2019/02/why-insect-populations-are-plummeting-and-why-it-matters/)

*Synopsis edited by: Rosario Marroquin-Flores (she/her/hers), PhD (Anticipated Spring 2022), School of Biological Sciences, Illinois State University.*

**Turning up the heat: how turtle hatchlings respond to environmental temperature**

**Featured Scientist:** Dr. Amanda Wilson Carter

**Birthplace:** New York City, NY

**My Research:** I study how animals are affected by changes in their environment.

**Research Goals:** I want to understand how changes in temperature affect animal **physiology**. I would like to be able to better predict how **climate change** will affect all types of animals.

**Career Goals:** I am currently working towards increasing the participation of underrepresented groups in science. My goal is to become a biology professor and researcher.

**Hobbies:** Traveling, playing with my dogs, and renovating my 1940’s bungalow.

**Favorite Thing About Science:** I love having the freedom to explore questions that I think are necessary and important. Throughout my schooling and career, I have also discovered a passion for mentoring students through independent projects in the lab. There is something very special about watching an undergraduate conduct their first study and transform into a confident and capable young investigator.

**Organism of Study:** I study many animals, but my PhD research focused on turtles, mainly the red-eared slider (***Trachemys scripta***). In my current position, I conduct research on dung beetles.

*Red-eared slider turtle (****T. scripta****). Image source*

**Field of Study:** Eco-**physiology** | **Climate Change** | Plasticity | Development | Parental Effects.

**What is Eco-physiology?** I broadly consider myself an eco- physiologist. I study how the **physiology** and behavior of animals are affected by their environment (mainly temperature).

**Check Out My Original Paper:** “Short heatwaves during fluctuating incubation regimes produce females under temperature-dependent sex determination with implications for sex ratios in nature”

*QR Code to the original publication*

**Citation: A.W. Carter**, B.M. Sadd, T.D. Tuberville, R.T. Paitz, R.M. Bowden, Short heatwaves during fluctuating incubation regimes produce females under temperature-dependent sex determination with implications for sex ratios in nature. *Sci. Rep.* 8.3: 1-13 (2018).

*Synopsis written by: Elyse McCormick, M.S. (Anticipated Spring 2022), School of Biological Sciences*, Illinois State University.

**Research at a Glance:** Temperature is very important for many parts of biology. For baby turtles, it is essential. Many turtles become male or female based on **incubation** temperature. This process is called **temperature-dependent sex determination**, or **TSD**. Warmer **incubation** temperatures make females, while cooler **incubation** temperatures make males. Until now, **TSD** has been studied in lab settings where the **incubation** temperature is held constant. However, since temperature doesn’t stay constant in nature, this is pretty unrealistic. Amanda wanted to see what happens to **sex ratios** when turtles experience real, changing temperatures. She hypothesized that changes in temperature are very important to determining sex in turtles that have **TSD**. She predicted that turtles are very sensitive to short exposures to temperatures above the “**pivotal temperature**”, or the temperature that will produce a population-wide 50:50 **sex ratio**. She also predicted that ovaries would start to grow within a few days of exposure to these warmer temperatures.

Amanda raised eggs using temperatures that went up and down with daily daytime and nighttime temperatures to mimic what a turtle would feel in nature. She then introduced eggs to short exposures of temperatures above the **pivotal temperature** to mimic heat waves. She was able to show that **sex ratios** can be changed by exposure to very short increases in temperature. Amanda used an Illinois population of the red-eared slider turtle, ***T. scripta***, for her research. Under the conditions in this study, ***T. scripta*** only needed approximately 8 days of exposure to warmer temperatures to produce a 50:50 **sex ratio**. At 5 days under the same conditions, 16% of the turtle **embryos** became female. This shows that even short exposures to warm temperatures can make an **embryo** become female. Amanda’s research shows that using natural temperatures can lead to a more accurate prediction of turtle **sex ratios** in nature. Her research also sheds light on another mystery. In Illinois, average temperatures are often so low that female turtles would be unlikely to be born. But somehow, females are still found in the population. Her research shows how female turtles end up in the Illinois population: turtle **embryos** only need a few days incubating under warmer temperatures to become female.

Amanda also wanted to see if **embryos** had the same response to temperature changes across the **nesting season**. She looked at differences between early and late season **embryos** to see how long it took under warmer temperatures to make female turtles. Late season **embryos** required less time at warmer temperatures to produce a 50:50 **sex ratio** than early season **embryos**. Mother turtles increase a hormone called **estrogen** in the **yolks** of their eggs across the **nesting season**, which leads to more females later in the season. Thus, late season **clutches** probably have a ‘head start’ on other **embryos** because they have more female **hormones** in their **yolk**. As a result, eggs don’t need as much time in warmer temperatures to make females. Amanda used the results from her experiments to then develop a mathematical model to better predict **sex ratios** using natural temperatures measured from the field. This model helps predict the number of male and female turtles based on real field temperatures.

**Highlights:** We just learned that females can be made after just a few days at warmer, **female-producing temperatures**. This finding helps us understand how sex is determined under more natural, changing temperatures. This finding is important because we can now more accurately determine **sex ratios** in turtle populations using available temperature datasets, like those available through the **National Oceanic and Atmospheric Association (NOAA)**. We are currently experiencing changing global temperatures, where turtle **embryos** are more likely to experience warm **incubation** conditions. Being able to predict **sex ratios** in turtles is important for their conservation. We might be able to accurately identify turtle species or populations that are most at risk of 100% female populations, which would be unable to make more offspring. We can use that information to direct conservation efforts and resources.

**What My Science Looks Like: Figure 1** from this paper is a great example of how different field temperatures can be. **Figure 1** reports temperature (y-axis) over the course of the summer **nesting season** (x-axis) for Illinois turtles. The solid horizontal line shows the **pivotal temperature** (**Tpiv**). It shows that, on average, temperatures have not been warm enough to produce females in this population for 23 years. But when you break it down by year, there are bursts of temperature warm enough to make females (see 1993 and 2012). In nature, temperature is not constant. Instead, it fluctuates. Amanda had to find a way to test what happens to turtle **embryos** in their natural environment. She needed temperature data from her study area to show that natural temperatures fluctuate around the **Tpiv**.

**Figure 1.** Average soil temperatures from Peoria, IL. *Figure adapted from Carter et al. 2018.*

**The Big Picture:** Amanda’s research took a more realistic look at **TSD**. It allowed her to create a mathematical model to predict realistic **sex ratios** for hatchling turtles. These types of studies allow scientists to better understand what’s happening in nature. Knowledge about how turtles live in the wild is important for more than just improving our understanding of turtle biology. They allow us to understand the wide-reaching effects of environmental impacts, such as **climate change**, on animals that are extremely sensitive to changes in temperature. **Climate change** could impact the **sex ratios** of hatchling turtles in ways we don’t fully understand yet. But this study allows us to start to understand it. Amanda’s model can be used to predict how **climate change** might affect future generations of turtles. It’s possible that her model can help us understand how **climate change** will affect turtles, as well as the animals and plants that they interact with. If we can understand how **climate change** might impact a broad array of plants and animals, we may be able to help lessen its effect on our environment.

**Decoding the Language:**

**Climate change:** A change in global or regional climate patterns, often seen as major changes in temperature or precipitation.

**Clutch:** A group of eggs that are laid together at the same time by a single female.

**Embryo:** An unborn or unhatched offspring that is actively growing. In the context of Amanda’s research, the embryo is the developing turtle inside the egg.

**Estrogen:** A steroid hormone that promotes the development and maintenance of female characteristics in the body.

**Female/Male-producing temperatures:** Female-producing temperatures are higher/warmer temperatures that cause red-eared slider turtles to become female. Male-producing temperatures are lower/colder and cause these turtles to become male.

**Incubation:** The process of keeping an egg warm enough to develop until hatching. In the context of Amanda’s research, a male-producing incubation temperature is approximately 26°C and a female-producing temperature is approximately 31°C.

**National Oceanic and Atmospheric Association (NOAA):** A scientific agency within the United States Department of Commence that focuses on oceans, major waterways, and the atmosphere.

**Nesting season:** The duration of time that animals are actively laying eggs. In the context of Amanda’s research, the nesting season lasts from late May to early July.

**Temperature-Dependent Sex Determination (TSD):** A form of sex determination where the incubation temperature determines whether the developing embryo will become male or female.

**Pivotal temperature (Tpiv):** The temperature that produces a population-wide 50:50 sex ratio.

**Physiology:** A branch of biology that studies how different parts of the body carry out chemical and physical functions.

**Sex ratios:** The amount of males compared to the amount of females, or vice versa.

***Trachemys scripta (T. scripta)*:** The scientific name for the red-eared slider turtle.

**Yolk:** The yellow-orange, nutrient-rich portion of the egg that supplies food to the developing embryo.

**Learn More:**

Illinois Turtles Fact Sheets (Illinois [DNR](https://www.dnr.illinois.gov/publications/%20Documents/00000699.pdf))

NASA’s climate model [site](https://climate.nasa.gov/)

*Synopsis edited by: Rosario Marroquin-Flores, PhD (Anticipated Spring 2022), School of Biological Sciences*, Illinois State University.

**Student Instructions**

**Reading:** Infection outcomes not significantly affected by thermal variability in a bumble bee host-parasite system (Author: Kerrigan Tobin)

**Discussion Forum on [LMS page]**

Read the attached PubNavigator article that highlights research done by Kerrigan Tobin, a former student in the Illinois State University School of Biological Sciences. After completing the reading, discuss your perceptions of the article with your discussion group. You may discuss anything that you find interesting relating to the article. Here are some possible discussion questions to get you started:

1. In order to conduct this experiment, what concepts did the scientists need to understand? What did they need to know about the species or system that they were researching? How did this knowledge base enable them to conduct the research?
2. In what ways might this experiment have been improved? Why/how would these improvements help us to gain a better understanding of the natural phenomena under study?
3. What did you find surprising or interesting about the results of this study?
4. After reading the article, are you still curious about Ecology or the Beneficial Acclimation Hypothesis? Why is this interesting to you?
5. How do you think the researcher feels about the work that they do?
6. How would you feel if you were to do this kind of research and why?
7. Were there things that you did not understand about the research? If so, try to help and answer each other’s questions.

**Your 1^st^ post will be** **due on Tuesday, [date]**, this will give each of you time to think about and respond to posts throughout the week. **All other posts will be due on Friday, [date]**. Each post should end with a question to help continue the conversation.

Regarding the content of your post, there is no minimum number of posts or words per post; your grade will be based on the thoughtfulness of your responses and your genuine engagement in the conversations about the article. Students who get full credit on this assignment engage in conversation every day and communicate with several, if not all, members of their group. Their posts put forth *new ideas* that have not been expressed in the conversation, and they respond to their peers’ posts with genuine reflection and consideration of their ideas. Statements made on the discussion board should be based in evidence, either from the article itself, or from an outside source^[1]^. See the attached grading rubric for more details on how your discussion will be graded.

It is expected that discussions in which people are genuinely engaged, there will be disagreement. This is typically a good sign because it shows people are thinking! But all disagreements should proceed respectfully, in which those in disagreement focus on the ideas being discussed while remaining respectful of the people discussing them.

**Instructor Notes:**

- Create an asynchronous discussion board on your LMS page using students in groups of 5-7
- The instructions above will be placed in the forum description

**Grading Rubric (15 points)**

**Initial Post** **(2 points)**

- Post is made on time (0.5 points)
- Post ends with a question (0.5 points)
- Post appropriately addresses the prompt (1 point)

**Engagement (5 points)**

- Engages with most, if not all, contributing students (2 points)
- Ends each post with a question to prompt further discission (1 point)
- Contributes to discussion with clear, concise comments on at least 4 of the 7 days that the assignment is open (2 points)

**Contribution (8 points)**

- Genuinely responds to posts (2 points)
- Contributes to and broadens the topic of discussion by posing new questions (3 points)
- Uses references to the literature, external readings, or personal experiences as evidence to support statements made on the discussion board^[2]^ (3 points)

**Sample Discussion Board**

**2021-21 (Feb 1, 2021 8:28 AM)** This article shed light on the important issue of global warming. I think when humans think about global warming we think of how it will effect the lives of other humans and we tend to spend less time thinking about the effect on native species. However, Kerrigan Tobin focuses on the effect of an average warmer temperature on a bees immune system. I had never heard of Beneficial Acclimation Hypothesis before, but now that I understand it, I would like to read more about how animals have already adjusted to rising global temperatures. There was one part of this article that I think could have been expanded on: using more than one kind of infection. I believe it would be interesting to test the efficacy of the bumblebees immune system against viruses and bacteria in different temperatures because the research showed no significant change in infection when using parasites. I was honestly surprised at the outcome of the research. How do you think the use of different types of pathogens would effect the outcome of a similar experiment?

**S2021-13(Feb 1, 2021 11:46 AM)** I really appreciate the perspective that you put the issue of global warming into where humans really only ever think about the effects that it will have on themselves, and not enough focus on other essential organisms on our planet. To answer your question, I think that by using different types of pathogens in a similar experiment, there could be a build up of more evidence that disproves the BAH. This could be valuable information in the long run. Another outcome could be that different pathogens serve as independent variables that the immune systems of the bees would process completely differently deepening on the pathogen/virus. Each pathogen could have unique effects.

[other discussion takes place]

**S2021-32 (Feb 2, 2021 8:08 PM)** Climate change, and specifically global warming are a threat to life as we know it. Most people don't realize how it could affect us, but it's because they don't realize that some species like bumblebees are the backbones to ecosystems. If one of them falls, the rest will collapse after. I agree it would be interesting to see the extreme temperature experimented with, and maybe see if there is any chance of some type of evolution possible. . . but hopefully we never get to that point.

**S2021-13 (Feb 3, 2021 11:13 AM)** I agree that it would be interesting to see how the bees handled parasite transmission at more extreme temperatures. My concern with that though is that if it is too extreme of a cold or hot temperature, it could interfere with the bee's health in different aspects than the parasite transmission. This could throw the experiment off because rather than observing how parasites are transmitted under different temperatures, the bees could actually die from being exposed to too much heat or cold and have other health issues. How do you think you could measure under more extreme temperatures without killing off the bees or posing other health issues that would distract from the intent of the experiment?

[other discussion takes place]

**S2021-32 (Feb 2, 2021 9:33 PM)** This study was honestly very interesting to me. Frankly, I was quite shocked by the results. It does give me some hope knowing that the bees did well in this experiment. However, what should be noted is that they were never put up against any extreme temperatures. The ones that were outside of their "comfort zone" were not out of it by a long shot. The temperature range for bees is when they're at their best, but that doesn't mean they can't function should the winter be a little colder one year or vise versa with a hotter summer than usual. This required a lot of information on the species and the invasive species. I'd say the main concept they needed to understand was how well the average bees immune system performs in conditions where its not necessarily in a comfortable environment. I think this study would've been better had they tested more bees and tested them in more harsh temperatures (on both the hot and cold spectrum). I've personally always been interested by ecology and my major is environmental sciences, so reading this article was genuinely interesting to me. If I was the researcher, I would be proud of this work; of course, there areas to improve on. I want to do this kind of research hopefully as soon as next summer. I am wondering if it would be possible to experiment with bees and see if they're capable of adapting to different temperatures over semi-short amounts of time (given that their lifespan isn't relatively long).

**S2021-13(Feb 3, 2021 11:24 AM)** I do agree that the results were almost somewhat expected in regards to the effect of temperature on the bees. My only concern with testing the bees in harsher temperatures is that they could be prone to more health problems from that in respect to them actually being able to survive and stay healthy. I think these added problems could distract from the focus on parasite transmission and data could be thrown by this. I think there could be a way to carry out what you're speaking of though and that this experiment is just serving as a good baseline to understand more and perform more complicated research in the future. Do you think it is possible for bees to acclimate to different temperatures that are more extreme without dying off or suffering health issues?

**Table 1.** Full codebook for argumentation and engagement.

| **Argument** | **Definition**: A passage of text that contains an assertion and a form of supporting or refuting evidence as justification for the statement. An argumentation unit minimally consists of a claim and supporting data, the facts used as the foundation for the claim but may also include a warrant (implicit rules or principles that connect the data to the claim), and/or backing (facts or assumptions that further support the warrant). | |
| --- | --- | --- |
| **Incomplete** | **Definition:** A statement made by a student that contains a claim but does not use data as justification for the stated claim. | **Example**: “I believe that the bees ability to resist the parasites are due to other factors that are not affected by the change in temperature. However it may also be that the temperature has not risen to the point that it affects their immune system. And the change in how the different hives are affected are caused by other reasons.” |
| **Complete** | **Definition:**  A statement made by a student that contains a claim and supporting data | **Example:** If there are any, TSD probably only affects a very small amount of species that aren't reptiles. However, I do think that reptiles are the main group affected by TSD due to them being cold-blooded organisms, meaning their body temperature is not regulated the same way ours or other warm-blooded organisms are.” |
| **Correct** | **Definition:**  A complete argument that contains scientifically accurate information. | **Example:** “I definitely think that if the turtles were to live in a wanner environment then the ratios would be different. In the lab, they had changed the temperature levels, which in turn changed the ratio, so you could assume that a much wanner environment would have more female turtles.” |
| **Incorrect** | **Definition:** A complete argument that makes scientifically inaccurate claim, or makes a claim based on incorrect information within the data, backing, and/or warrant used to justify the claim. | **Example:** “I think to answer your question, since there are so many crickets in the world and in the wild I believe inbred breeding does happen more frequently than outbred and inbred breeding. I think this is so due to information based off Kylie's study that she provided. Crickets in the wild in populations live and grow with crickets of their own family. Therefore from this study it shows they will prefer to mate with their own rather than mating with crickets outside of their inbred family.” |
| **Simple** | **Definition:**  A complete argument that contains a claim and on sources of data to justify the claim. | **Example**: “I think this is a really interesting question because there are so many different ways that bees impact the environment. For example, they are necessary for pollination. The possibility that they might be impacted negatively by climate change would be detrimental to the plant life surrounding their colonies.” |
| **Complex** | **Definition:** A complete argument that includes a warrant, backing and/or multiple forms of data to support the claim. | **Example:** “I highly agree with you [student name], since inbred crickets are incapable of breeding properly and developing at a "normal" rate. I can see them going extinct because of this, like both [student name] and I think the extinction of crickets would heavily affect the food web. As we discussed in another post crickets are usually food for many amphibians and reptiles, they also eat other insects, the transition between eating crickets to another insect would not be as difficult as it seems.” |
| **Engagement** | **Definition**: A statement, or several statements, that indicate that the student is engaged with the reading. | |
| **Connecting to the Scientist** | **Definition:** A statement, or several statements, where a student describes their thoughts about the focal researcher, including their perceptions of the focal researcher’s emotions, actions, and/or knowledge. | **Example:** “I feel as though she is very passionate in her research and really does enjoy what she is doing.” |
| **Importance of the Research** | **Definition:** A statement, or several statements, where a student states that they think the research is important and/or describes why the research is important. | **Example:** “After reading about the different conditions under which turtle sex is determined, a few key concepts jumped out at me. The first was that an external stimulus could have an effect on sex, such as temperature. This factor is important because global warming could quickly create a threat to turtle populations.” |
| **Critique** | **Definition:** A statement, or several statements, where a student shares ways they think the research and/or article could be improved. | **Example:** “One way that the experiment could be improved is to create an outside environment that can be used to test the natural temperate changes that occur in nature, instead of re-creating the temperatures in a lab. This could give more realistic results on how the eggs are aﬀected in nature.” |
| **Interest in the Research** | **Definition:** A statement, or several statements, where a student expresses that they found the research interesting. | |
| **General Interest** | **Definition:** A statement, or several statements, where a student expresses a general interest in the research using words, such as, “interesting”, “fascinating”, and “surprising”, or expressed general excitement about the research | **Example:** “I thought the most interesting bit of information in this study was that embryos only need 5 days of warm temperatures to become female. It’s such a short period of time, its honestly quite amazing.” |
| **Desire to Participate** | **Definition:** A statement, or several statements, where a student expresses a desire to participate in the research or in research similar to the research described in the article. | **Example:** “I believe that other animals that have genders based on temperature include crocodilians, many reptiles, and some fish. This means that the rising temperatures would not only affect the turtle populations negatively. Due to this, I would love to complete research on one or more animals in order to further investigate this issue.” |
| **Interest in the Research** | **Definition:** A statement, or several statements, where a student expresses that they found the research interesting. | **Example:** “I found this article very interesting and informative. Temperature plays a big factor in determining the sex of turtles, which I find very fascinating. One thing I would like to continue to research on the topic is the male determination.” |
| **Relating to Outside Knowledge** | **Definition:** A statement, or several statements, where a student relates concepts discussed in the article to outside knowledge and/or information. | |
| **Relating to Other Biological Concepts** | **Definition:** A statement, or several statements, where a student relates a biological concept in the article to another biological concept. | **Example:** “The decrease in the number of turtles is startling, and I wonder how it will affect the ecosystem. I believe that, without their predation, the number of small fish will grow rapidly, depleting their own food sources, in turn hurting them. Also, any creatures that prey on turtles will have less to eat, possibly declining their numbers. In the long run, I see an ecosystem crash where populations get smaller and smaller.” |
| **Relating to Other Species** | **Definition:** A statement, or several statements, where a student relates a concept about a species discussed in the article to another species. | **Example:** “I am currently into reptiles at the moment, and I know that there are two factors in determining the gender. The first is sex chromosomes, which is like how mammals are determined. Snakes and lizards are also determined this way. The other factor is by temperature, like turtles. There are a couple other reptiles that are determined this way, which are alligators and crocodiles.” |
| **Relating to Personal Experiences** | **Definition:** A statement, or several statements, where a student relates a scientific concept from the article to their personal experience, such as, through anecdotes about experiences with friends or family, regional experiences, or about experiences in the classroom. | **Example:** “This makes me think of how in Texas this year, when they had very cold weather, and had to put turtles indoors. I wonder if that has impacted any of the turtles’ eggs' sex, or if it was not detrimental because it was not breeding season.” |

1. Recommended language to be added to student instructions
2. Recommended language to be added to the grading rubric
